# A new era of natural killer cell immunotherapy in tumor treatment: latest advances and cutting-edge perspectives from basic research to clinical practice

**DOI:** 10.3389/fimmu.2026.1795143

**Published:** 2026-04-29

**Authors:** Xin Du, Shixin Shi, Haiyan Liu

**Affiliations:** 1 Department of Nuclear Medicine, First Hospital of Shanxi Medical University, Taiyuan, China; 2 Collaborative Innovation Center for Molecular Imaging of Precision Medicine, Shanxi Medical University, Taiyuan, China; 3 China Atomic Energy Authority (CAEA) Center of Excellence on Nuclear Technology Applications for the Diagnosis, Treatment & Transformation of Nuclear Medicine, Taiyuan, China

**Keywords:** cancer, cancer immunotherapy, CAR NK cell therapy, chimeric antigen receptor, NK cell, tumor microenvironment

## Abstract

As early as 1863, Virchow observed that cancer often arises at sites of chronic inflammation. Modern epidemiological and clinical studies have confirmed the link between inflammation and cancer. Natural Killer (NK) cells actively participate in and regulate inflammatory processes; however, they are not strictly classified as classic ‘inflammatory cells’ in cellular taxonomy. NK cells rapidly identify and eliminate malignantly transformed cells in a non-major histocompatibility complex (MHC)-restricted manner, a characteristic that distinguishes them from other immune cells. Furthermore, their use in allogeneic settings carries a very low risk of graft-versus-host disease (GvHD), making them ideal candidates for developing ‘off-the-shelf’ cellular immunotherapies. Although early clinical attempts using unmodified NK cells showed limited efficacy, the past decade has witnessed rapid advancements in genetic engineering, cell expansion and differentiation, and synthetic biology, propelling NK cell therapy into a new era of development. This article aims to provide a systematic and multi-dimensional review of the latest research progress in NK cell therapy. We begin by revisiting the core biological basis of NK cell anti-tumor activity, focusing on design strategies, clinical breakthroughs, and bottlenecks of Chimeric Antigen Receptor NK (CAR-NK) cell therapy in hematological malignancies and solid tumors. We delve into antibody-based NK cell recruitment strategies (such as BiKEs/TriKEs) and techniques to enhance antibody-dependent cellular cytotoxicity (ADCC), and analyze cytokine-induced memory-like NK (CIML-NK) cells as a non-gene editing enhancement strategy. Simultaneously, we focus on the core challenges currently faced by NK cell therapies, particularly in solid tumors, including poor tumor infiltration, potent suppression by the tumor microenvironment (TME), and limited *in vivo* persistence. We summarize diversified synergistic strategies, such as combination with immune checkpoint inhibitors, radiotherapy, chemotherapy, targeted drugs, and direct modifications of the TME. Finally, this article discusses contentious points within the field and provides a forward-looking perspective on future directions, striving to offer a comprehensive and insightful reference for the translation of NK cell therapy from the laboratory to widespread clinical application.

## 1 Introduction

Cancer remains one of the most rapidly evolving global health challenges. According to World Health Organization data, 2022 saw an estimated 20 million new cancer cases and 9.7 million cancer deaths worldwide. Demographic projections suggest that by 2050, the number of new cancer cases annually will reach 35 million, a 77% increase from the 2022 figure (1). Traditional treatment modalities, primarily surgery, radiotherapy, and chemotherapy, have saved countless lives in the past. However, their non-specific “collateral damage” (“killing one thousand enemies while losing eight hundred soldiers”) and issues of tumor drug resistance and recurrence have limited further progress in clinical treatment (2). Since the 21st century, cancer immunotherapy has brought new dawn to cancer treatment. Immune checkpoint inhibitors (ICIs) and Chimeric Antigen Receptor T-cell (CAR-T) therapy, as landmark treatments (3), work by reactivating the body’s own immune system to attack tumors and have achieved success in treating various malignancies. With broader clinical application, the limitations of these two therapies have also become apparent. The efficacy of ICIs varies greatly among different cancer types, showing good response primarily in immunogenic “hot tumors” such as melanoma and lung cancer (4). CAR-T therapy achieves high cure rates in B-cell hematological malignancies but has limited efficacy in solid tumors, accompanied by potentially fatal severe adverse effects like cytokine release syndrome (CRS) and immune effector cell-associated neurotoxicity syndrome (5). More importantly, currently approved CAR-T products are all autologous, requiring weeks for preparation, costing hundreds of thousands of dollars, and some patients cannot undergo production due to poor quantity or function of their own T cells (6), all of which limit the widespread adoption of this “personalized customization” therapy. Against this backdrop, researchers have turned their attention to another crucial force in the immune system-Natural Killer cells. As the “first responders” of innate immunity, NK cells possess unique advantages: (1)Non-MHC-restricted killing: NK cells recognize target cells through the integration of signals from their surface activating and inhibitory receptors, enabling them to effectively eliminate tumor cells that downregulate MHC-I molecules to evade T-cell attack (“missing-self” recognition). (2) Low GvHD risk from allogeneic sources: Infusion of allogeneic NK cells rarely causes GvHD, facilitating the development of universal or “off-the-shelf” cell products. (3) Diverse killing mechanisms: Beyond direct cytotoxicity, NK cells can secrete cytokines like IFN-γ to regulate and mobilize adaptive immune responses, and can mediate antibody-dependent cellular cytotoxicity via their CD16 a receptor, synergizing with antibody drugs (7–10). This article aims to provide a comprehensive overview and outlook on NK cell immunotherapy. We will trace its development history, detail the current cutting-edge engineering strategies and clinical applications, objectively assess the challenges and controversies, and based on this, conduct an in-depth discussion on future trends and potential technological breakthroughs, striving to provide a valuable reference for researchers and clinicians in this field.

## 2 Basis of NK cell anti-tumor activity

### 2.1 NK cell recognition mechanism: the dynamic balance of activation and inhibition

NK cell function is regulated by a sophisticated “signal integration model.” Their cell membrane is populated with various activating and inhibitory receptors, and the final cellular response depends on the net balance of signals transmitted by these receptors (11–13). The main families of inhibitory receptors on the NK cell surface include Killer-cell Immunoglobulin-like Receptors (KIRs) and C-type lectin-like receptors (such as CD94/NKG2A) (14). These specifically recognize classical (HLA-A, B, C) and non-classical (HLA-E) MHC class I molecules, which are ubiquitously expressed on healthy cells (15). This recognition transmits an inhibitory “off” signal to the NK cell, preventing attack on normal tissue. However, during evolution, tumor cells often downregulate or completely lose MHC-I expression to evade Cytotoxic T Lymphocyte (CTL) recognition and attack. This very behavior relieves the inhibition mediated by KIRs/NKG2A, allowing NK cells to recognize and eliminate these “disguised” abnormal cells according to the “missing-self” hypothesis (16). The main activating receptors on the NK cell surface include Natural Cytotoxicity Receptors (NCRs, such as NKp30, NKp44, NKp46), NKG2D, and DNAM-1, among others (16). The ligands for these receptors are typically expressed at low levels or not at all on normal cells but are significantly upregulated when cells undergo stress stimuli such as viral infection, DNA damage, or malignant transformation. For example, ligands for NKG2D (MICA/B and ULBPs) and DNAM-1 (PVR/CD155 and Nectin-2/CD112) are classic “stress-induced ligands” (17). When NK cells encounter tumor cells expressing high levels of these ligands, activating signaling pathways are triggered, initiating the killing program. This “induced-self” recognition mechanism is key to NK cell surveillance of tumors (2, 18, 19).

#### 2.1.1 Cytotoxic mechanisms of NK cells

NK cells primarily exert their cytotoxic effects through three classical mechanisms. The granule exocytosis pathway is the most classic and efficient means of killing: upon forming an immunological synapse with target cells, NK cells directionally release cytotoxic granules containing perforin and granzymes. Perforin forms transmembrane pores in the target cell membrane, allowing granzymes (primarily granzyme B) to enter the cytoplasm and rapidly induce target cell apoptosis by activating the caspase cascade (20–22). The death receptor pathway serves as a complementary mechanism, whereby activated NK cells express death receptor ligands such as FasL (CD178) and TRAIL (Apo2L), which bind to their corresponding receptors Fas (CD95) and TRAIL-R1/R2 on the surface of target cells, thereby directly initiating the extrinsic apoptotic pathway (23). Antibody-dependent cell-mediated cytotoxicity (ADCC) involves NK cells that highly express the activating Fc receptor CD16a (FcγRIIIa) (24). When tumor-specific antibodies (such as rituximab or trastuzumab) bind to tumor cells via their Fab fragments, the Fc fragments are recognized by NK cell CD16a, potently activating NK cells and mediating target cell lysis. ADCC is one of the core mechanisms underlying the clinical efficacy of numerous therapeutic monoclonal antibodies (25). Together, these three mechanisms constitute the molecular basis by which NK cells recognize and eliminate abnormal target cells.

#### 2.1.2 Secretion of cytokines and chemokines

**Figure 1:**
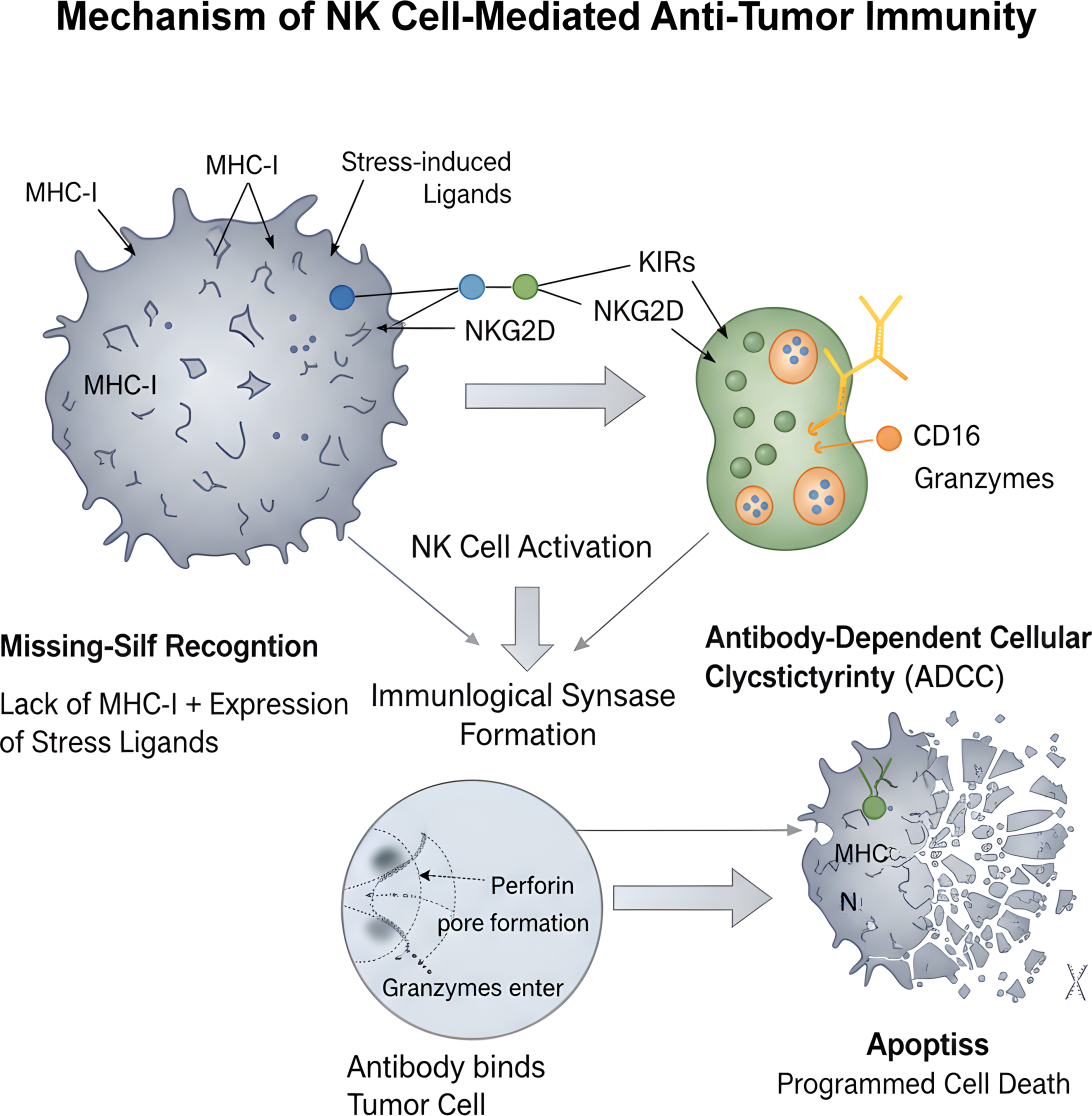
Schematic illustration of natural killer (NK) cellmediated antitumor immunity. NK cells can be activated via “missing self recognition” (downregulation of MHCI and upregulation of stressinduced ligands on tumor cells) or through ADCC, leading to immunological synapse formation, release of perforin and granzymes, and induction of tumor cell apoptosis.

Activated NK cells are important sources of immunomodulatory factors, possessing both pro-inflammatory and immunoregulatory functions. They secrete large amounts of cytokines such as IFN-γ, TNF-α, GM-CSF, G-CSF, and chemokines like CCL3, CCL4, CCL5, XCL1 (26). IFN-γ can not only directly inhibit tumor cell proliferation and angiogenesis but, more importantly, can upregulate MHC-I expression on tumor cells (enhancing T cell recognition), promote Dendritic Cell (DC) maturation, and recruit and activate other immune cells like T cells (27). Thus, NK cells effectively bridge innate and adaptive immune responses, reshaping the entire tumor immune microenvironment (**Figure 1**).

## 3 NK cell therapy

NK cell therapy has evolved from simple cell infusions to a modern, multi-technology integrated precision treatment system. This includes strategies such as CAR-NK engineering, genetic editing optimization, combination therapies, exploration of metabolic regulation mechanisms, and immune checkpoint inhibition. Each approach has unique benefits and limitations, requiring personalized treatment plans based on cancer type, stage, and patient health status. The continuously evolving landscape of these therapies continues to advance cancer treatment options.

### 3.1 CAR-NK cell therapy

The advent of chimeric antigen receptor technology has made immunotherapy a revolutionary approach in the field of cancer treatment. CAR-T therapy, initially successfully applied to T cells, has achieved significant progress in the treatment of B-cell lymphomas and leukemias (28–31). However, this therapy faces several bottlenecks, including the lengthy time required to produce therapeutic doses and the difficulty in obtaining sufficient autologous T cells from heavily pre-treated cancer patients (32). CAR-NK cell therapy has emerged as a potential solution to the challenges faced by CAR-T therapy. A CAR is an engineered receptor protein, typically composed of a single-chain variable fragment connected via a hinge region to a transmembrane domain, followed by an intracellular co-stimulatory domain and a CD3ζ signaling domain (33). CAR-engineered NK cells not only enable precise recognition of specific tumor antigens but also fully retain the inherent biological characteristics of NK cells (34). Functionally, CAR-NK cells preserve the rapid killing mechanisms of innate immune cells (such as the perforin-granzyme mediated apoptosis pathway), low immunogenicity, and ADCC effects, while gaining the ability to specifically recognize tumor antigens through the CAR structure, thereby significantly reducing the risk of non-specific damage to normal cells (35, 36).

Compared to CAR-T cells, CAR-NK cell therapy offers significant advantages in terms of safety. The incidence of severe cytokine release syndrome and neurotoxicity associated with CAR-NK cells is extremely low. Activated NK cells primarily secrete IFN-γ and GM-CSF, whereas CAR-T cells can induce a variety of cytokines related to CRS (such as IL-1, IL-2, IL-6, IL-8, IL-10, and TNF-α). As T cells recognize host tissues through their receptors, allogeneic CAR-T cells carry a significant risk of inducing graft-versus-host disease (GvHD); however, CAR-NK cells do not express the T cell receptor, thus posing a very low risk of causing GvHD. “Off-the-shelf” CAR-NK products can be safely applied without the need for patient-specific matching. More importantly, CAR-NK cells can utilize multiple complementary target recognition mechanisms and can identify and eliminate cancer cells through CAR-independent pathways, including natural cytotoxicity receptors (NCRs), NKG2D, DNAX accessory molecule-1 (DNAM-1), and activating killer cell immunoglobulin-like receptors (KIRs), as well as CD16-mediated ADCC (37–39). This multi-pronged recognition strategy effectively reduces the risk of tumor escape due to antigen loss, a challenge that is particularly common in CAR-T cell therapy.

#### 3.1.1 CAR-NK structure design and optimization

Early CAR-NK studies often directly adopted CAR constructs originally designed for T cells. However, the intrinsic activation mechanisms, signal transduction pathways, and biological functions of these two immune cell types are fundamentally distinct (40). Optimization of T-cell CARs focuses on mimicking and enhancing the T-cell receptor (TCR) signaling pathway, utilizing classical costimulatory domains such as CD28 or 4-1BB to regulate T-cell metabolism, proliferation, and memory differentiation. In contrast, optimization of NK-cell CARs must return to the inherent activation logic of NK cells, incorporating NK-specific signaling molecules such as DAP10, 2B4, and DNAM-1, while meticulously tailoring the matching of transmembrane domains and signaling domains (41).

##### 3.1.1.1 Extracellular antigen-binding domain

CARs designed for T cells utilize a single-chain variable fragment (scFv) as the antigen-binding domain, composed of the light and heavy chains of a monoclonal antibody linked by a flexible peptide linker (42). However, the scFv structure has inherent limitations: the light and heavy chains are prone to misfolding and aggregation, resulting in suboptimal structural stability. This not only compromises antigen recognition efficiency but can also induce antigen-independent T-cell activation, leading to premature T-cell exhaustion. For NK cells, incorporating the extracellular domain of a natural NK cell activating receptor, such as NKG2D, into the CAR structure-while not a conventional “CAR” (as NKG2D is not an scFv)-can leverage the intrinsic recognition mechanisms of NK cells for improved functionality (43).

##### 3.1.1.2 Hinge region and transmembrane domain

The hinge region and transmembrane domain connect extracellular antigen recognition to intracellular signal transduction and critically influence CAR expression levels, spatial conformation, and signaling thresholds. In T-cell CAR designs, the length and flexibility of the hinge region require precise tuning: a hinge that is too short may cause steric hindrance, affecting antigen binding, while one that is too long may induce nonspecific activation. The transmembrane domain is often derived from CD8α or CD28; the latter can also form heterodimers with endogenous CD28 molecules to enhance signal transduction. For NK cells, when the NKG2D transmembrane domain is used in CAR design, although it can recruit endogenous DAP10 for signal transduction, its functional efficacy is not optimal (44). In contrast, a sequential combination of the CD28 transmembrane domain and the DAP10 signaling domain has demonstrated enhanced cytotoxic effects in NK cells. The design of the transmembrane domain in NK-cell CARs must consider compatibility with endogenous signaling adaptor molecules, rather than simply transplanting mature components from T-cell CARs.

##### 3.1.1.3 Intracellular signaling domain

The design of intracellular signaling domains in T-cell CARs follows the T-cell receptor activation mechanism. First-generation CARs contain only the CD3ζ chain, which provides activation signals but lacks co-stimulatory function; second-generation CARs introduce one co-stimulatory domain (typically CD28 or 4-1BB); third-generation CARs incorporate two co-stimulatory domains (e.g., CD28 + 4-1BB) (45). Among these, CD28 recruits kinases such as PI3K, GRB2, and LCK through its intracellular YMNM, PRRP, and PYAP motifs, activating the PI3K/AKT pathway to promote glycolytic metabolism and effector function, thereby significantly enhancing T-cell expansion and persistence. In contrast, 4-1BB activates the NF-κB pathway via TRAF adaptor proteins, favoring the formation of memory T cells (46).

However, for NK cells, directly adopting T-cell CAR structures may result in suboptimal activation. NK cell activation does not rely on TCR-like signaling but rather on adaptor molecules such as DAP10, DAP12, 2B4, and DNAM-1 to transmit activation signals. A comparative study of seven CAR structures in NK cells further confirmed that CAR architecture directly influences *in vivo* persistence and anti-tumor efficacy (47). For example, anti-CD5 CAR-NK cells expressing the 2B4 signaling domain exhibited stronger cytotoxicity and *in vivo* anti-tumor activity than those expressing the T-cell-associated co-stimulatory domain 4-1BB. Therefore, developing NK cell-specific combinations of signaling domains is critically important.Based on these characteristics, the intracellular signaling domain design of CAR-NK cells is more distinctive. First-generation CAR-NKs utilize only the ITAM signaling domain (primarily CD3ζ) for primary signaling without co-stimulatory molecules; second-generation CAR-NKs incorporate one intracellular co-stimulatory domain; third-generation CAR-NKs integrate two or more co-stimulatory domains. Commonly used co-stimulatory domains that enhance cytotoxic potential and promote cytokine production include CD28, 4-1BB, 2B4(CD244), CD27, NKG2D, and DAP-10/12. Fourth-generation and fifth-generation CARs are built upon the second-generation platform. Fourth-generation CAR-NKs act as transgenic cytokine carriers, releasing cytokines such as IL-7, IL-12, IL-15, IL-18, IL-23, or combinations thereof, thereby enhancing anti-tumor immune responses through cytokine secretion and supporting CAR-NK cell survival (42). Fifth-generation CAR-NKs incorporate STAT sequences and carry intracellular cytokine receptors (e.g., the IL-2 receptor IL-2R), which can enhance cell activation and proliferation (44). Beyond cytokine genes, incorporating suicide genes into the CAR structure can also enhance anti-tumor efficacy and enable controlled activity regulation. Notably, NK-CAR12, constructed by sequentially combining DAP10 (instead of the NKG2D transmembrane domain) with the 2B4 signaling domain and CD3ζ, demonstrated superior cytotoxicity and anti-tumor efficacy compared to conventional T-cell CARs in a CD19-positive lymphoma model (48), providing a new direction for the optimization of CAR-NK signaling domain design.

#### 3.1.2 NK cell sources and manufacturing platforms

Current CAR-NK production primarily relies on the following cell sources: Peripheral Blood (PB-NK) is widely available, with established collection methods allowing large-scale collection from healthy donors and isolation via immunomagnetic beads or flow cytometry. The advantage is that their physiological state is close to *in vivo* NK cells; the disadvantage is significant inter-donor variability, and *in vitro* expansion and transduction efficiency are noticeably affected by donor differences. Umbilical Cord Blood (CB-NK) is a core platform for early CAR-NK as an allogeneic “off-the-shelf” product, offering advantages like easy cryopreservation and weaker HLA restrictions. The CD19 CAR-NK study reported by Liu et al. in NEJM used CB-NK as the starting cell source. NK cell lines (e.g., NK-92) offer advantages such as strong proliferation capacity and relatively stable phenotype, suitable for standardized production and repeated freeze-thaw cycles. However, they require irradiation before infusion to reduce the risk of tumorigenicity, resulting in limited *in vivo* persistence. Induced Pluripotent Stem Cell (iPSC)-derived NK (iPSC-NK) enables a clonal, infinitely expandable single-cell source, facilitating fully standardized GMP production, and is one of the most rapidly developing platforms recently. CAR and other functional genes can be integrated during early differentiation stages of iPSC-NK, allowing for multi-gene editing and batch-to-batch consistency control (49).

#### 3.1.3 Breakthroughs of CAR-NK in hematological malignancies

With advancements in CAR structure optimization, diversification of cell sources (peripheral blood, cord blood, NK cell lines, iPSC-derived NK), and strategies such as IL-15 armoring, genetic editing to regulate cell metabolism and immune checkpoints, CAR-NK has achieved significant progress in B-cell malignancies, acute myeloid leukemia, and multiple myeloma (50). Currently, the most representative clinical evidence for CAR-NK comes from the phase I/II trial of cord blood-derived CD19 CAR-NK reported by Liu et al. in NEJM. This study enrolled 11 patients with relapsed/refractory CD19-positive lymphoma or CLL, who received HLA-mismatched CB-NK cells transduced with a retroviral vector to express a CD19 CAR, IL-15, and iCasp9. Patients received only a single infusion, with no observed grade ≥3 CRS, neurotoxicity, or GvHD. The objective response rate was approximately 73%, with a complete response rate of about 64%. Most responses were achieved within 30 days post-infusion, and some patients maintained long-term remission (34). Subsequent expanded cohorts based on similar platforms and studies from other centers have confirmed the potential of CD 19 CAR-NK in B-cell non-Hodgkin lymphoma and B-ALL (51). However, compared to B-cell malignancies, CAR-T development for Acute Myeloid Leukemia and Multiple Myeloma itself faces challenges such as difficult target selection and significant overlap between myeloid/plasma cell targets and normal hematopoietic/immune cells (52). CAR-NK, with its more controllable toxicity and shorter *in vivo* persistence, is theoretically considered a more suitable treatment approach for these diseases.

#### 3.1.4 Bottlenecks of CAR-NK in solid tumor treatment

The dense extracellular matrix (ECM) and rich vascular network of solid tumors form a substantial physical barrier (53–55). Additionally, the mismatch between the chemokine receptor profile of NK cells and the chemokines present in the tumor locale is a major reason why CAR-NK cells struggle to effectively accumulate in the tumor area (56). The expression level of target antigens varies greatly in different regions of solid tumors, and some tumor cells may not express the target antigen at all, leading to antigen-negative relapse after CAR-NK attack. The solid tumor TME is rich in soluble factors that inhibit NK cell activation, such as TGF-β, PGE2, IDO-derived metabolites, adenosine, and lactate (57). It is also accompanied by the accumulation of myeloid-derived suppressor cells, regulatory T cells, and M2-type tumor-associated macrophages, forming a multi-layered immunosuppressive network (58). In this context, NK cells often upregulate inhibitory receptors like NKG2A, KIR, TIGIT, and PD-1, while tumor cells and TME cells highly express ligands such as HLA-E and PD-L1, leading to the suppression of CAR-mediated signaling and the emergence of an exhausted phenotype (59). This functional impairment not only weakens immune surveillance but is also closely related to tumor progression and metastasis. A 2023 single-cell data analysis of 716 patients published in Cell showed a significant correlation between the functional state of NK cells and the risk of tumor metastasis, confirming the clinical necessity of deeply understanding TME suppression mechanisms (60). Understanding the complex characteristics of the TME, particularly metabolic reprogramming within this environment, holds significant clinical importance for developing effective treatments for cancer patients (61).

##### 3.1.4.1 Immunosuppressive factors inhibit the function of NK cells

The solid tumor microenvironment is enriched with immunosuppressive factors like TGF-β, PGE2, IDO-derived metabolites, and high concentrations of adenosine, which can significantly downregulate NK activating receptors (e.g., NKG2D, NKp30, CD16) and inhibit degranulation and IFN-γ production. TGF-β, a cytokine produced by tumor cells, Tregs, MDSCs, and other stromal cells in the TME, can inhibit the expansion and function of effector cells and promote Treg proliferation, impairing NK cell function through various pathways (62, 63). TGF-β inhibits the transcription factor T-bet (via SMAD3) and downregulates the expression of NKp30, NKG2D, and its ligands MICA, thereby limiting NK cell cytotoxicity and IFN-γ production (64). Interestingly, TGF-β can transdifferentiate NK cells into ILC 1-like cells that lack cytotoxic function (65). Based on these findings, TGF-β can be considered a target for enhancing NK cell-mediated anti-tumor immunity. Prostaglandin E2 in the TME, produced by tumor cells, TAMs, and stromal cells, is a key regulator of NK cell activity. PGE2 binds to EP2 and EP4 receptors on NK cells, engaging the common cAMP-PKA signaling pathway (66), thereby reducing cytotoxicity (67). The intracellular enzyme Indoleamine 2, 3-dioxygenase, a key regulator in the TME, converts tryptophan to L-kynurenine (68). L-kynurenine affects NK cell activity by interfering with IL-2-driven upregulation of NKp46 and NKG2D (69). IDO overexpression is associated with tumor progression and the growth arrest of tumor-infiltrating NK cells. Adenosine inhibits the maturation and effector functions of NK cells by binding to the A2AR receptor, further reducing their anti-tumor efficacy (70).

##### 3.1.4.2 Hypoxia inhibits the antitumor activity of NK cells

Hypoxia is a notable feature of the solid tumor microenvironment, with oxygen concentrations < 1% found in 90% of solid tumors. This environment significantly suppresses the anti-tumor activity of NK cells; studies show that NK cell activity is almost completely abolished in hypoxic regions. The high energy demands of tumor cells lead to oxygen deprivation, placing infiltrating NK cells in a hypoxic state, which subsequently remodels their functional phenotype through Hypoxia-Inducible Factor-1α (HIF-1α)-mediated metabolic reprogramming (71). Under hypoxia, HIF-1α expression is upregulated in NK cells, driving a metabolic shift from oxidative phosphorylation to glycolysis via its target genes, including BNIP3, PDK1, VEGF, PKM2, and LDHA. *In vitro* studies show that exposing NK cells to a hypoxic environment(1.5% O_2_) stabilizes HIF-1α expression, which can upregulate glycolysis-related enzymes (e.g., LDHA) and glucose transporters (e.g., GLUT1) to meet energy demands in hypoxia (72). The impact of this metabolic shift on NK cell function is cell state-dependent: resting NK cells exhibit reduced proliferation capacity and impaired anti-tumor activity after hypoxic exposure; whereas NK cells pre-activated by 7–9 days of normoxic culture can, under hypoxia, coordinate the regulation of cell cycle genes (upregulating CCNE1, CDC6) and cytotoxicity-related genes via the HIF-1α/ERK/STAT3 pathway, demonstrating enhanced tumor-killing capacity (71). The role of HIF-1α in NK cells exhibits a significant duality. On one hand, HIF-1α expression in tumor-infiltrating NK cells correlates negatively with anti-tumor function, and inhibiting HIF-1α can enhance degranulation and the production of IFN-γ and TNF-α in human NK cells. On the other hand, in viral infection models, HIF-1α-deficient NK cells showed a significant reduction in cell numbers due to impaired glucose metabolism and increased expression of the pro-apoptotic protein Bim, failing to effectively control viral load (73). This paradox may be related to the activation state of NK cells and the specific microenvironment: freshly isolated peripheral blood NK cells cannot stabilize HIF-1α under hypoxia, leading to impaired killing function; whereas expanded NK cells can restore killing activity in hypoxic environments by upregulating HIF-1α target genes (74).

### 3.2 Gene editing technologies

NK cells are highly dependent on IL-15. Traditional allogeneic NK cell infusions often rapidly decline *in vivo* due to the lack of sustained cytokine support. Integrating membrane-bound or secreted IL-15 into the CAR construct can maintain a metabolic and survival advantage for NK cells within the local microenvironment. Liu et al.’s cord blood CD19 CAR-NK co-expressed IL-15 and achieved detectable *in vivo* expansion without additional systemic IL-2/IL-15 administration. Subsequent research found that IL-15 signaling itself triggers the upregulation of negative regulators like CISH, limiting NK cell efficacy. Knocking out CISH significantly enhanced the metabolic fitness and persistence of IL-15-armored CAR-NK cells. In long-term, low-cytokine *in vitro* cultures and leukemia xenograft models, CISH-/- CAR-NK cells exhibited stronger killing activity and *in vivo* persistence. Traditional CAR optimization often relied on empirical selection of candidate genes. Recent genome-wide CRISPR screening efforts in primary human NK cells have identified several key factors closely related to killing capacity and metabolic homeostasis, such as MED12, ARIH2, and CCNC. Knocking out these genes significantly enhanced the cytotoxicity, cytokine secretion, and metabolic activity of CAR-NK cells (75, 76). Building on this, multi-gene editing strategies are emerging. For example, simultaneously knocking out CISH and CBLB to relieve multiple negative regulatory circuits; or achieving triple knockout of CD70/CISH/CBLB in CD70-targeting CAR-NK cells, enabling the cells to maintain high killing capacity even in inhibitory microenvironments like those containing TGF-β (42). These findings hold significance not only for AML and myeloma, which reside in complex bone marrow microenvironments, but also for solid tumors.

### 3.3 Combination therapy strategies

Combination therapies, by relieving tumor microenvironment suppression or enhancing NK cell effector function, significantly expand the therapeutic window. PD-1 inhibitors combined with NK cells show synergistic effects in solid tumors. For instance, SMT-NK (allogeneic NK) combined with Pembrolizumab in chemotherapy-resistant advanced biliary tract cancer achieved a disease control rate (DCR) of 73.9%, a median progression-free survival of 4.1 months, and a 70.4% reduction in liver metastases in a 76-year-old patient (77). A new model of NK cell pre-activation with bispecific antibodies: a 2025 Nature Medicine publication on a phase I trial (NCT04074746) of AFM13 (a CD30/CD 16A bispecific antibody) pre-activated NK cells for refractory relapsed lymphoma showed an ORR of 92.9%, CR rate of 66.7%, 2-year overall survival rate of 76.2%, with 11 patients maintaining CR for 14–40 months. The core mechanism involves the bispecific antibody simultaneously engaging CD16A on NK cells and CD30 on tumors, bridging effector and target cells and relieving immune suppression (78).

#### 3.3.1 Combination with bispecific NK cell engagers

Bispecific NK cell engagers (BiKEs) are recombinant protein molecules composed of two single-chain variable fragments (scFvs), with one end recognizing tumor antigens and the other end binding to the CD16a receptor on the surface of NK cells, thereby activating endogenous NK cells to exert ADCC effects through cross-linking (79). The advantages of BiKEs include the absence of cellular engineering requirements, relatively controllable production costs, and applicability as standardized protein drugs; their small molecular structure may confer favorable biodistribution properties, particularly suitable for solid tumor therapy. However, BiKEs face limitations such as short half-life requiring frequent administration, dependence on the functional status of endogenous NK cells, and inability to form immunological memory (80). BiKEs and NK cells are not mutually exclusive; the combination of BiKE-secreting CAR-T cells with exogenous NK cells exerts synergistic anti-tumor effects in both hematological and solid tumor models, while reducing the dose dependency on CAR-T cells, thereby achieving optimized therapeutic efficacy through the complementarity of these two treatment modalities (81).

#### 3.3.2 Combination with immune checkpoint inhibitors

Exhausted NK cells in the TME also highly express immune checkpoint molecules such as PD-1, TIGIT, and TIM-3, while tumor cells and other inhibitory cells in the TME (like MDSCs) express ligands like PD-L1 and PVR (the TIGIT ligand) to suppress NK cell function (82). Therefore, ICIs can not only “release the brakes” on T cells but also on NK cells. Numerous preclinical studies and ongoing clinical trials indicate that NK cell therapy combined with anti-PD-1/PD-L1 antibodies can produce synergistic anti-tumor effects (83).

#### 3.3.3 Combination with traditional radiotherapy and chemotherapy

Traditional therapies are no longer viewed merely as “cell killers” but also as “modulators” of the immune system. Many chemotherapy drugs (e.g., anthracyclines, cyclophosphamide) and radiotherapy can induce “immunogenic cell death” (ICD) in tumor cells (84). During ICD, tumor cells release Damage-Associated Molecular Patterns (DAMPs, such as ATP, HMGB1) and upregulate the expression of molecules like NKG2D ligands (MICA/B), Fas, and TRAIL-R on their surface, making them more susceptible to NK cell recognition and killing. In summary, after radiotherapy/chemotherapy reduces tumor burden and creates an “immune-friendly” window, NK cell infusion can achieve a multiplier effect.

#### 3.3.4 Combination with targeted drugs

Certain small-molecule targeted drugs can also produce unexpected synergistic effects with NK cell therapy. For example, proteasome inhibitors (e.g., Bortezomib), used in treating multiple myeloma, were found to upregulate the expression of the TRAIL receptor DR5 on myeloma cells, thereby enhancing NK cell killing via the death receptor pathway. Epigenetic modulators, such as HDAC inhibitors, can also increase tumor cell susceptibility to NK cells by remodeling chromatin and upregulating molecules like NKG2D ligands (85).

#### 3.3.5 Integrated multi-strategy iPSC-NK platforms

The iPSC-NK platform offers the ultimate possibility for “built-in” combination strategies. Taking Fate Therapeutics’ star product FT596 as an example (86), it integrates three functional modifications into one iNK cell: (1) A CD19-CAR for direct targeting of B-cell malignancies; (2) A high-affinity, non-cleavable CD16 (hnCD 16) (87), enabling efficient synergy with CD20 monoclonal antibodies like Rituximab via ADCC to kill tumors; (3) An IL-15/IL-15Rα fusion protein (IL-15RF), serving as an autocrine growth factor to support *in vivo* proliferation and survival without exogenous cytokines. Such a product constitutes a powerful combination therapy itself, targeting multiple antigens (CD19 + CD20) via multiple mechanisms (CAR + ADCC). Its Phase I clinical trial in B-cell lymphoma demonstrated excellent efficacy and safety, representing the future direction of “off-the-shelf” cell therapies (88).

### 3.4 Cytokine-induced memory-like NK cells

A significant discovery is that briefly “pre-activating” NK cells *in vitro* with a specific cytokine combination (e.g., IL-12, IL-15, and IL-18 for about 16 hours) can induce their differentiation into a subset with adaptive immune memory characteristics, known as Cytokine-Induced Memory-like NK (CIML-NK) cells (89). These cells undergo epigenetic and transcriptional reprogramming. Upon re-encountering tumor stimuli, they produce much larger quantities of IFN-γ and have a longer *in vivo* survival period. After infusion in AML patients, CIML-NK cells can persist for months or even over a year, whereas conventionally expanded NK cells typically last only one to two weeks. Finally, they do not require sustained cytokine support post-infusion, eliminating the need for high-dose IL-2. In a clinical trial of relapsed/refractory acute myeloid leukemia (AML), the infusion of CIML-NK combined with chemotherapy achieved an overall response rate of 55% (5/9) and a complete response rate of 45% (4/9), demonstrating significant clinical potential (90, 91). CIML-NK opens a new and efficient pathway for non-genetically engineered NK cell therapy (92).

## 4 Controversies, challenges, and future expectations for NK cell therapy

### 4.1 Controversies and challenges

The use of CAR-T/NK/M cell therapies for cancer treatment faces multiple challenges, particularly the difficulty in target selection due to tumor heterogeneity and the immunosuppressive nature of the tumor microenvironment. Future research should focus on exploring methods to alleviate the immunosuppressive effects of the TME, identifying more specific and practical tumor-associated antigens, and developing safer and more effective CAR vectors to better harness the biological functions of NK cells. Research on GPC3 CAR-NK cells in hepatocellular carcinoma treatment has progressively accumulated substantial preclinical data (93–96), moving beyond initial proof-of-concept studies (97, 98). The fourth-generation GPC3 CAR-NK cells developed by Busà et al. through genetic engineering have garnered significant attention (99). By co-expressing IL-15 and IFN-α, these cells exhibit markedly enhanced *in vitro* cytotoxic activity and cytokine release. Additionally, the incorporation of a truncated EGFR as a suicide gene provides a product design that balances functionality and safety for subsequent clinical translation. As research progresses, the limitations of single-target GPC3 therapy have gradually become apparent. The immunosuppressive tumor microenvironment, heterogeneous expression of the GPC3 antigen, and insufficient penetration of CAR-NK cells into solid tumor tissues all constrain therapeutic efficacy. Consequently, studies have explored combination strategies, pairing GPC3 CAR-NK cells with local treatments such as radiotherapy or ablation (100, 101). These approaches leverage synergistic mechanisms to relieve local immunosuppression while improving immune cell infiltration and persistence within the tumor region. Future research should prioritize optimizing these combination regimens to enable more precise and safer application tailored to individual patient characteristics.

As a current research hotspot, CRISPR-Cas9 can modify the inherent defects of NK cells at the genetic level while amplifying their advantages, thereby enabling NK cells to better exert their functions, achieve improved therapeutic outcomes, and reduce treatment risks for patients (75). In terms of checkpoint gene disruption, tumor cells often exploit inhibitory receptors to evade immune attack. Using CRISPR-Cas9 to introduce frameshift mutations or premature stop codons into the NK cell genome can block the expression of specific inhibitory receptors (such as PD-1, TIGIT, and LAG-3) or their downstream signaling molecules; knockout of CISH relieves the negative feedback inhibition of the IL-15 signaling pathway, significantly enhancing the metabolic activity and proliferative capacity of NK cells; upon tumor activation or cytokine stimulation, PD-1 expression in NK cells increases from low to high levels, and PD-1 knockout prevents tumor cells from inducing NK cell dysfunction via PD-L1 (102, 103). In terms of enhancing cytokine signaling, NK cells are prone to apoptosis and exhibit poor *in vivo* persistence without exogenous IL-2 support. Following knock-in of an IL-15/IL-15Rα fusion protein via gene editing (104), NK cells autonomously express membrane-bound IL-15, providing an autocrine survival signal, which greatly enhances their survival advantage and eliminates dependence on exogenous cytokine infusion. In terms of countering immunosuppressive signals, the tumor microenvironment is rich in inhibitory molecules such as transforming growth factor-β (TGF-β) and prostaglandin E2. TGFBR2 is the receptor for the immunosuppressive molecule TGF-β; after CRISPR-mediated knockout of TGFBR2, NK cells maintain their cytotoxic function even when exposed to high concentrations of TGF-β in the tumor microenvironment (97). By leveraging CRISPR-Cas9 technology to delete inhibitory genes that restrict NK cell activity or introduce genes that enhance their survival, proliferation, and cytotoxic function, the suppressive effects of the tumor microenvironment can be overcome, thereby achieving better therapeutic outcomes. We believe that future research should focus on the flexible integration of CRISPR-Cas9 systems in combination with other well-established approaches to more effectively harness NK cell functions and develop therapeutic strategies tailored to individual patient characteristics (86, 105).

How long NK cells persist in the host to enhance clinical efficacy remains an unresolved question. This necessitates in-depth research into the correlation between the peak expansion of NK cells and treatment outcomes. Furthermore, NK cell persistence relies on cytokine support, and exogenous cytokines might cause adverse side effects, such as the growth of Tregs (95). Additionally, understanding how immunosuppressive factors in the TME, like low glucose, hypoxia, and certain cell populations (mainly MDSCs, Tregs, DCs, and TAMs), inhibit the anti-tumor function of lymphocytes is crucial. Insufficient solid tumor infiltration and a lack of long-term safety data remain major bottlenecks. The future requires the development of “TME-adapted NK cells” synergistically optimized through chemokine receptor engineering (e.g., CALHM2 knockout to enhance infiltration) and metabolic enhancement (e.g., IL-15RF fusion), to achieve targeted colonization of the TME and functional persistence. Currently, studies show that the distribution of NK cell subsets varies with tumor type and does not strictly correlate with their distribution in the blood, while also discovering unique NK cells within the TME (106). This could become the next hotspot for research. Therefore, I believe that using single-cell sequencing to characterize different NK cell subsets is crucial. Systematic classification will lead to a more comprehensive understanding of NK cells, enabling personalized cancer treatment and bringing new hope to cancer patients.

### 4.2 Development opportunities for NK cell therapy

Designing more intelligent, logic-gated CAR-NKs, for instance, requiring the simultaneous recognition of two antigens for activation, or activating an inhibitory signal upon recognition of normal tissue markers, thereby greatly improving targeting precision and safety (107, 108).

Establishing global, standardized “iPSC Master Cell Banks” to significantly reduce the production cost of iPSC-NK, making “off-the-shelf” NK cell therapy a truly affordable and globally accessible medicine.

Utilizing high-throughput analyses like single-cell sequencing and spatial transcriptomics on patient tumor tissues to precisely map the immune landscape of their TME. Spatial transcriptomics is a technology that integrates high-throughput sequencing with *in-situ* spatial information. It can analyze the whole transcriptome expression profile while preserving the original structure of tissues and map it to the precise spatial coordinates of tissue sections. In the research of NK cell immunotherapy, the application value of spatial transcriptomics is mainly reflected in four aspects. First, it can analyze the cell homing mechanism, identify the spatial distribution of NK cells in tumor tissues and the specific anatomical locations where they are excluded (such as perivascular areas or tumor stroma), and reveal the physical barriers that limit infiltration (109). Second, it can identify functional subsets. By integrating spatial localization and transcriptional features, it can identify functional NK cells enriched in specific niches. For example, the SPON2^+^ NK cell subset at the tumor invasive front is significantly associated with improved patient prognosis and can serve as a biomarker for predicting treatment efficacy (110). Third, it can analyze the interaction network of the microenvironment. Based on the co-expression of ligand-receptor and cell co-localization patterns, it can reconstruct the *in-situ* communication between NK cells and fibroblasts, macrophages, and tumor cells, and clarify the formation mechanism of the immunosuppressive niche (111). Fourth, it can guide cell engineering modification, providing a theoretical basis for the gene editing strategy of NK cells. For example, it can enhance directional migration by knocking in chemokine receptors or design new CAR structures based on functionally identified molecules (such as SPON2) (110). In summary, the core value of spatial transcriptomics lies in establishing the spatial association between the molecular phenotype of NK cells and the tissue microenvironment, enabling researchers to understand the functional regulation mechanism of NK cells from a multi-dimensional *in-situ* perspective and providing a theoretical basis for optimizing the next-generation engineered NK cell treatment strategies for solid tumors. Based on this, the most suitable combination treatment can be “tailor-made” for the patient. For example, selecting CAR-NK cells expressing a dominant-negative TGF-β receptor for tumors with active TGF-β signaling.

Interestingly, the powerful anti-viral and immunoregulatory capabilities of NK cells also show great potential in non-oncological areas such as treating chronic viral infections, autoimmune diseases, and suppressing organ transplant rejection (112). Taking the field of autoimmune diseases as an example, Jie Gao et al. found in a clinical trial of CD19 CAR-NK therapy for systemic lupus erythematosus (SLE) that after more than 12 months of follow-up in 18 patients, 67% achieved DORIS remission and 76% achieved LLDAS (113). This represents an important new blue ocean for the future development of NK cell therapy.

## 5 Conclusion

NK cell therapy represents a milestone development in the field of cancer treatment, offering new hope for improving patient response rates, prolonging survival, and enhancing quality of life. This review analyzed the latest advances in NK cell therapy and the bottlenecks in solid tumor treatment. From the excellent safety and efficacy demonstrated by CAR-NK in hematological malignancies, the “off-the-shelf” industrial future heralded by the iPSC-NK platform, to the intrinsic memory potential revealed by CIML-NK, all these approaches have yielded encouraging results in pre-clinical and clinical trials. However, there are also significant challenges that cannot be ignored, including tumor heterogeneity, the nature of the tumor microenvironment, and the risk of immune evasion. Furthermore, comprehensive evaluation of cell product manufacturing processes, patient selection criteria, and the long-term safety and efficacy of these therapies is crucial. We have reason to believe that NK cell therapy could become a powerful weapon for curing malignant tumors, bringing a new dawn for cancer patients.
